# Bioethanol production from alkali-pretreated rice straw: effects on fermentation yield, structural characterization, and ethanol analysis

**DOI:** 10.3389/fbioe.2023.1243856

**Published:** 2023-08-03

**Authors:** Reema Ningthoujam, Pankaj Jangid, Virendra Kumar Yadav, Dipak Kumar Sahoo, Ashish Patel, Harish Kumar Dhingra

**Affiliations:** ^1^ Department of Biosciences, School of Liberal Arts and Sciences (SLAS), Mody University of Science and Technology, Lakshmangarh, Rajasthan, India; ^2^ Department of Life Sciences, Hemchandracharya North Gujarat University, Patan, Gujarat, India; ^3^ Department of Veterinary Clinical Sciences, College of Veterinary Medicine, Iowa State University, Ames, IA, United States

**Keywords:** bioconversion, biofuels, pretreatment, fermentation, biomass, rice straw

## Abstract

Current ethanol production technology has a dire need for efficient conversion of lignocellulosic biomass to fermentable sugars. The conversion requires pretreatment of the biomass, one of the most expensive steps, and thus it is quite necessary to identify the most cost-effective and high-efficiency conversion method. In this study, rice straw (RS) biomass was pretreated using 4% NaOH alkali, soaked for 4 h, and autoclaved for 30 min. The structural and morphological changes were examined using Fourier transform infrared spectroscopy (FTIR), X-ray diffraction (XRD), and scanning electron microscopy (SEM) analysis in both native and alkali-treated RS. The FTIR analysis revealed that native RS contains a considerable amount of lignin that was removed after the pretreatment process. The XRD pattern of the RS revealed an increasing crystallite size of the pretreated lignocellulosic biomass. The study of SEM clearly showed the distorted structure and surface porosity after the pretreatment process. Enzymatic hydrolysis efficiency was checked by comparing the commercial enzymes and microbial hydrolysis extracted from a fungal isolate. The best-reducing sugar yield obtained was 0.62 g/L, achieved at optimized conditions from the commercial enzymes. Fermentation efficiency was checked using the yeast isolate *Saccharomyces cerevisiae* in both the native and pretreated substrate, and the highest ethanol concentration (21.45%) was achieved using 20% w/v biomass loading, enzyme loading (2:1:1), and fermentation for a week at 30°C and pH 4.5. This concentration was higher than that of the untreated RS (3.67%). The ethanol thus produced was further checked for analysis by the ^1^H and ^13^C nuclear magnetic resonance (NMR) methods.

## 1 Introduction

The increase in population has led to increased energy consumption, causing a shortage of fossil fuels, in turn driving a dire need to develop alternative energy sources (Mankar et al., 2021). Ethanol obtained from renewable resources has been of great interest in the past few decades (Taghizadeh-Alisaraei et al., 2019). Bioethanol production utilizing biomass consisting of cellulosic components is a cleaner and safer choice than fossil fuels, which are non-renewable energy resources (Sanni et al., 2022). Currently, bioethanol is most commonly produced using first-generation conversion technologies that rely on food-based agricultural crops such as sugars or starch (Melendez et al., 2022). At present, bioethanol production from lignocellulosic wastes, which are termed second-generation technologies, has become an alternative way as these methods do not compete with food crops or agricultural land, are economical, abundantly available, and have lower transportation costs (Yadav et al., 2022). Low-cost ethanol can be produced utilizing lignocellulosic biomass; hence, bioethanol produced from biomass is considered an attractive, renewable energy source for fuel transportation (Broda et al., 2022).

Regarding total production, one of the major staple crops produced globally is rice, mostly grown in Asia and viewed as the world’s third most important grain crop (Fukagawa Naomi and Ziska, 2019). Rice crop cultivation is accompanied by the production of rice straw (RS), an agricultural waste, and the options for disposal are directly burning it in the fields (Bhattacharyya et al., 2021). The degradation rate in the soil is slow and might enhance stem-rot disease in rice. Burning is a major practice that releases smoke and creates pollution, consequently affecting the environment and public health. RS is characterized by having low alkali and high cellulose (Sahoo et al., 2013) and hemicellulose (Huang et al., 2021; Guleria et al., 2022; Kaur et al., 2023) content that can convert to fermentable sugars, which is why it is considered an attractive feedstock for bioethanol production (Singh et al., 2021). In these waste products, the lignin acts as a barrier and provides resistance against enzymatic action, while the cellulose and hemicellulose are intertwined with each other and packed densely in the cell wall of the plant (Goodman, 2020; Kumari and Singh, 2022). The carbohydrates should be made easily accessible for further hydrolysis processes by disrupting the lignin. Thus, a pretreatment step is necessary when converting the lignocellulosic biomass to high-degree fermentable sugars (Laca et al., 2019). However, the composition of RS varies depending upon several parameters, such as climatic conditions, type of soil, genetic variability, and environmental influences (Takano and Hoshino, 2018; Khantibongse and Ratanatamskul, 2023). If efficiently used, RS can be utilized as a feedstock for the competitive production of bioethanol, resulting in lessening fossil fuel dependence and further reducing pollution. An estimated 650–975 million tonnes of RS are produced annually worldwide (Bhattacharyya et al., 2020; Hassan et al., 2021). China alone produces 400 million tonnes of straw that could, in fact, be used as a replacement for 200 million tonnes of coal (Hassan et al., 2021). A recent study unveiled annual RS production of 370–520 million tonnes worldwide, 330–470 million tonnes in Asia, and 100–140 million tonnes in Southeast Asia (Ren et al., 2019; Van Hung et al., 2020; AboDalam et al., 2022).

The overall conversion of lignocellulosic biomass to bioethanol consists of three major steps: pretreatment, hydrolysis of enzymes, and lastly, fermentation. The pretreatment step is the most crucial as it greatly impacts the overall bioconversion and causes changes in the microstructure, macrostructure, and chemical composition of the lignocellulosic biomass. In the case of substrates such as RS, pretreatment is quite necessary to achieve the best yield of fermentable sugar (Malik et al., 2022; Deng et al., 2023). The aim of pretreatment is to disrupt the recalcitrant nature and increase in surface area of the cellulosic biomass, making cellulose more accessible to enzymes so that it can easily convert carbohydrates into fermentable sugars and remove hemicelluloses and lignin. However, eliminating lignin and hemicellulose depends on the types of pretreatment techniques, severity, and process conditions (Zhao et al., 2020).

There are several reported pretreatment methods, *viz.*, the physical, chemical, physico-chemical, and biological, as well as their combinations. Chemical pretreatment can easily digest the enzymatic saccharification process and increase the yield of fermentable sugars (Sunkar and Bhukya, 2022). However, using inorganic acids, such as HCl and H_2_SO_4_, often leads to severe corrosion of the equipment and excessive degradation of the carbohydrates. Therefore, selecting a mild pretreatment method that is eco-friendly can improve the overall economic performance of biofuels (Luo et al., 2022; Luo et al., 2023). Moreover, physicochemical technologies have been extensively researched to demonstrate their distinct mechanisms of action for breaking down cell walls. Currently, studies are being made on converting biomass to fermentable sugars using microorganisms such as cellulose-degrading fungi, which are found to be environmentally friendly and less expensive (Tan et al., 2021; Ningthoujam and Dhingra, 2022). Ningthoujam and Dhingra (2021) also demonstrated the viability of *Trichoderma reesei* and *Aspergillus flavus* as cellulase producers (Ningthoujam and Dhingra, 2021). A single pretreatment method is considered ineffective; therefore, efforts are being made to study various combination methods that can produce higher theoretical yields (Dimos et al., 2019; Khan et al., 2021). Luo et al. (2022) stated that the addition of detoxified reagent enhanced the enzymatic hydrolysis of the pretreated biomass (Luo et al., 2022).

In the present investigation, native RS was selected as a feedstock for bioethanol production. Changes in functional groups, changes in crystallite size, and structural changes in RS were studied both in pretreated RS and after pretreatment. The study was investigated by using Fourier transform infrared spectroscopy (FTIR), X-ray diffraction (XRD), and scanning electron microscopy (SEM). In addition, the fermentation efficiency of both the native and pretreated RS was checked. The ethanol thus obtained was determined using the ^1^H and ^13^C nuclear magnetic resonance (NMR) spectroscopy techniques.

## 2 Materials and methods

### 2.1 Raw materials and chemicals used

The RS was collected from a nearby field in Imphal East District, Manipur, India. The RS was dried, chipped, and ground to size particles less than 1 mm. The finely powdered RS was thoroughly washed with double distilled water (ddw) to eliminate all the soluble contents present in it. The rinsing with distilled water continued until the wash water became clear. The commercial cellulase enzyme (ONOZUKA R-10) and *α*-amylase (from Malt) were obtained from HIMEDIA, Mumbai, India; amyloglucosidase obtained from *Aspergillus niger* was procured from SIGMA, United States. Furthermore, the cellulolytic fungus *Aspergillus flavus* (Accession No. OK330386) was isolated from a soil sample to check its cellulase activity. *Saccharomyces cerevisiae* (Accession No. OK189580), which is ethanologenic, was isolated from grapes to check its fermentation efficiency.

### 2.2 Lignocellulosic biomass pretreatment

The NaOH pretreatment of RS was performed in a conical flask loaded with 10% biomass (w/w) with 4% (w/v) NaOH, soaked for 4 h and incubated at room temperature, that is, 27°C, and then autoclaved at 121°C and 15 lb pressure for 30 min. Highly alkaline residues (approx. pH 10–12) were washed with distilled water until they attained pH 7.0, filtered, and dried for 48 h at 65°C. A measure of 0.96 g of the substrate was reduced after the washing process, and this pretreated substrate was later used for enzymatic hydrolysis. Alkaline pretreatment, compared to acidic and hydrothermal processes, formed fewer inhibitory compounds; thus, no exogenous reagents were added to remove the inhibitors.

### 2.3 Compositional analysis of the substrate

For the compositional analysis, cellulose, hemicellulose, and lignin were analyzed as per Yang et al. (2006). Hemicellulose content was determined by taking 1 g of RS and treating it with 150 mL of 0.5 M NaOH aqueous solution, and incubating it at 80°C for 3.5 h. Again, it was extensively washed to eliminate the presence of Na^+^ ions, maintained the pH at 7.0, and dried to a constant weight. Hemicellulose content was considered the difference in weight before and after the RS treatment. Lignin content was measured by taking hemicellulose-free RS (0.5 g) treated with 98% sulfuric acid (15 mL) and incubated for 2 h at 30°C, then diluted to 4% sulfuric acid using de-ionized water. It was then autoclaved for 1 h at 121°C. Titration with 10% BaCl_2_ was performed to remove the sulfate ions present in the remaining RS, and the sample was dried in a hot air oven for 24 h at 65°C. The final weight obtained was estimated as the lignin content. Finally, $Cellulose\;content=Total\;content-\left(Hemicellulose+Lignin\right)$ (Xin and Geng, 2010).

### 2.4 Hydrolysis of enzymes

In this step, the pretreated substrate underwent enzymatic saccharification, performed by incubating 10 g of pretreated RS in 50 mM citrate buffer and maintaining the pH at 4.8. The enzymatic hydrolysis was carried out using commercial cellulase enzyme (ONOZUKA R-10), *α*-amylase from Malt (HIMEDIA), and amyloglucosidase solution from *Aspergillus niger* (Sigma Aldrich).

Enzymatic hydrolysis by the enzymes, as mentioned previously, was checked in different random combination ratios (0:1:1, 1:0:0, 1:0:1, 1:1:1, 2:1:1, and 1:2.5:1), and the best combination ratio producing the maximum reducing sugar was identified. The microbial enzymatic activity of two microorganisms, the strain *Trichoderma reesei* (MTCC 4876), which is one of the most famous producers of cellulases, and the novel fungal isolate *Aspergillus flavus* (Accession No. OK330386), which was isolated from the soil sample, was compared. Both samples, each containing one of the two microorganisms, were incubated at 45°C for 96 h at 150 rpm in order to assess their enzymatic hydrolysis activity. The reduced sugar obtained was quantified using the previously described method (Dsouza et al., 2012).

### 2.5 Structural characterization of rice straw

#### 2.5.1 FTIR analysis

FTIR spectra were analyzed to investigate the changes in functional groups in both the untreated and alkaline-treated biomass. FTIR analysis was performed using the PerkinElmer spectrophotometer model RX I (V max in cm^−1^) using KBr and was recorded at 400–4,000 cm^−1^ absorption band mode.

#### 2.5.2 XRD analysis

Untreated and alkaline-treated RS samples were analyzed by XRD instrument PANalytical X'Pert Pro. The crystallite size denoted by ‘D’ was calculated using the following equation: $D=K\times \frac{\lambda }{\beta }\mathrm{Cos}\theta ,$ where D (hkl) = size of the crystallite (nm); k = Scherer constant; *λ* = X-ray wavelength; *β* = full-width at half-maximum of the reflection hkl measured at 2θ, which is the corresponding Bragg angle (Sunkar and Bhukya, 2022).

#### 2.5.3 SEM analysis

Changes in the surface morphology of the RS after alkaline pretreatment were observed by SEM and were compared with the native biomass. The surface morphology images of both the untreated and chemically treated substrate were taken using a scanning electron microscopy Model JSM 6100 (JEOL) with SEM Image Analyser software.

### 2.6 Yeast strain, inoculum preparation, and fermentation conditions

Fermentation products were checked using a yeast strain isolated from the skin of grapes and were found to be *Saccharomyces cerevisiae* (Accession No. OK189580), which was identified by sequencing its regions using the primers ITS1 5′TCC​GTA​GGT​GAA​CCT​GCG​G 3′ and ITS4 5′TCC​TCC​GCT​TAT​TGA​TAT​GC 3'. It was then submitted to GenBank as a new yeast strain. The inoculum was prepared by growing it in YEPD media and incubating it at 30°C for 24 h. The isolate extracted from the sugar-rich habitat was found to be more productive than the commercial strain.

The hydrolyzed RS suspended in citrate buffer was used as the fermentation medium throughout the study. Simultaneous saccharification and fermentation (SSF) were selected for fermentation along with the ethanologenic yeast isolate. The medium was inoculated with 1 × 10^8^ cells/mL with a biomass concentration of 10% (w/w). The fermentation conditions were a) fermentation time (24, 48, 72, and 96 h), b) pH (4, 4.5, 5, 6, and 7), c) temperature (25°C, 27°C, 30°C, 35°C, and 40°C), and d) concentration of inoculum (2.5%, 5%, 7.5%, and 10%). At the end of each fermentation experiment, the broth was centrifuged at 6,000 rpm for 10 min, and ethanol concentrations were estimated by the potassium dichromate method **(Generalic, Eni. 2023**).

### 2.7 Ethanol analysis

The chemical structure of the extracted compounds was determined by observing the ^1^H NMR and ^13^C spectra. The ^1^H and ^13^C NMR spectra were recorded using DMSO solvent at 500 MHz on a Bruker Advance NEO spectrometer. The ^1^H NMR (proton nuclear magnetic resonance) technique is one of the quantitative determination methods for ethanol, and tetramethylsilane (TMS) is used as a reference. This chemical shift is represented by ‘δ’ and is considered to be 0.0 ppm. It is an accurate and rapid method for determining the presence of ethanol. Furthermore, the use of NMR over GC or HPLC for ethanol analysis is preferred due to its non-destructive nature and minimum sample requirement. The simplified proton NMR spectrum of ethanol enables the hydrogen atoms to be easily identified. ^13^C or Carbon-13 nuclear magnetic resonance, or carbon NMR, allows the identification of carbons present in ethanol.

## 3 Results and discussion

### 3.1 Compositional analysis of the rice straw

After pretreatment of the feedstock with alkali, its lignocellulosic composition was measured. The lignocellulosic contents of the untreated and treated RS were compared and are shown in Table 1. The cellulose content after NaOH pretreated RS increased to 45%. Pretreatment also resulted in increasing the accessibility to cellulose, and also, during enzymatic hydrolysis, it yielded maximum fermentable sugar. The hemicellulose content was found to be reduced to 20% in pretreated biomass, whereas it was 25% in native biomass. The reason may be because of its amorphous structure in nature that easily hydrolyzes during the chemical treatment. Almost all the lignin content was removed, falling from 17% in native biomass to 4% in treated biomass. Earlier, Sindhu et al. (2012) also analyzed the chemical constituents of RS and reported that cellulose was approximately 34 ± 3.5%, hemicellulose was approximately 28.45 ± 3.2%, and total lignin was approximately 18.12 ± 3.02%. There is a slight composition variation of the RS in our result obtained compared to that obtained by Sindhu et al. (2012). It may be due to different soil composition/texture, temperature, humidity, or variety of the rice. However, our obtained result was in close agreement with Sindhu et al. (2012).

**TABLE 1:** Composition of cellulose, hemicellulose, and lignin content before and after pretreatment of rice straw biomass.

Rice straw	Cellulose (%)	Hemicellulose (%)	Lignin (%)
Native	29	25	17
Alkaline pretreated	45	20	4

### 3.2 Enzymatic hydrolysis

Glucose was the standard sugar used for comparison with the pretreated rice RS hydrolyzate. Production of glucose was only observed in pretreated biomass. An optimized combination ratio of 2:1:1 was used for the final enzymatic hydrolysis. The optimum conditions for enzymatic hydrolysis in our present investigation were biomass loading (10 g), enzyme loading (2:1:1), and incubation time of 96 h at 45°C, which releases a maximum reducing sugar of 0.62 g/L. Even after enzymatic hydrolysis, the untreated biomass produced no reducing sugar, revealing that pretreatment is strictly required to convert the biomass to sugars (Mankar et al., 2021). Both commercial enzymes and microorganisms known to produce cellulolytic enzymes were used in our study to compare their efficiency. The actions of microbial enzymatic hydrolysis were checked, and both organisms tested were found to produce cellulase enzyme; however, this process took relatively longer than commercial enzymes to convert the biomass to reducing sugars.

### 3.3 Surface characterization of the rice straw

#### 3.3.1 FTIR analysis of pretreated and untreated rice straw for functional group identification

**FIGURE 1:**
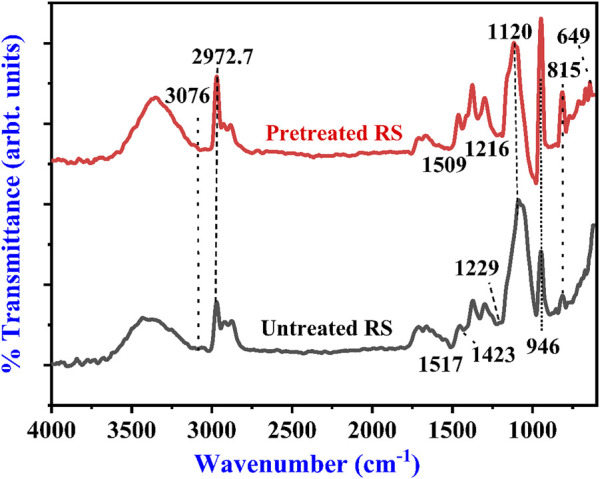
FTIR spectra of untreated and alkali-pretreated rice straw.

The structural difference in the untreated and alkali-treated RS was revealed by the FTIR spectra. Figure 1 clearly shows the changes in the spectra of treated and untreated RS with regard to intensity and shape. The bands of both samples were compared, and it was found that some of the band positions were altered, and some bands were absent in the pretreated RS due to alkali treatment. The broad band at 3,600–3,200 cm^−1^ corresponds to the O-H stretching of hydrogen bonds, and the signal at 2,972.7 cm^−1^ has been assigned to the C-H bonds, which are the characteristic features of cellulose (Bhattacharyya et al., 2020; Mankoo et al., 2023; Yan et al., 2023). This band was reduced after the NaOH treatment, indicating that methyl and methylene of cellulose had some rupture. The bands at 1,643.2 and 1,634.7 cm^−1^ are associated with deformation vibrations of H-OH in absorbed water. The band near 1,423 cm^−1^ can represent C-H bending in cellulose and hemicellulose (Md Salim et al., 2021). The band at 1,120 cm^−1^ indicates C-O stretching at C-3, C-O stretching at C-6, and C-C stretching (Arun et al., 2020). In addition, the band at 1,517 cm^−1^ is attributed to the C=C stretching of the aromatic ring of lignin (Prajapati and Kango, 2023). The band value of lignin ranges from 1,542 to 1,484 cm^−1^ (Arun et al., 2020; De et al., 2020; Sreejith et al., 2022) and was absent in the pretreated RS**.** Sreejith and team also obtained similar results for the RS in the range of 750–1800 cm^−1^ (Mankar et al., 2021). Table 2 shows the major FTIR assignments of the untreated and treated RS.

**TABLE 2:** Major FTIR assignments of untreated and alkali-pretreated rice straw.

Wavenumber (cm^−1^)	Functional group assignment	Related biomass component	Reference
3,600–3,200	O-H stretching of H bond	Cellulose	Phitsuwan et al. (2017)
2,972	C-H stretching	Cellulose	He et al. (2008)
1,643	C=C aromatic skeletal vibrations	Lignin	He et al. (2008)
1,517	C=C stretching	Lignin	Louis et al. (2022)
1,423	C-H deformation	Cellulose	He et al. (2008)
1,120	Si-O-Si	Silicates	Yadav and Fulekar (2019); Yadav et al. (2023)
1,059	C-O stretching/Si-O-Si		Kaur and Kuhad (2019)
1,260	Guaiacyl ring C-O stretching	Change in lignin monomer	Sonwani et al. (2020)
815	C-H out of plane	Cellulose	He et al. (2008)

#### 3.3.2 XRD analysis for phase identification

**FIGURE 2:**
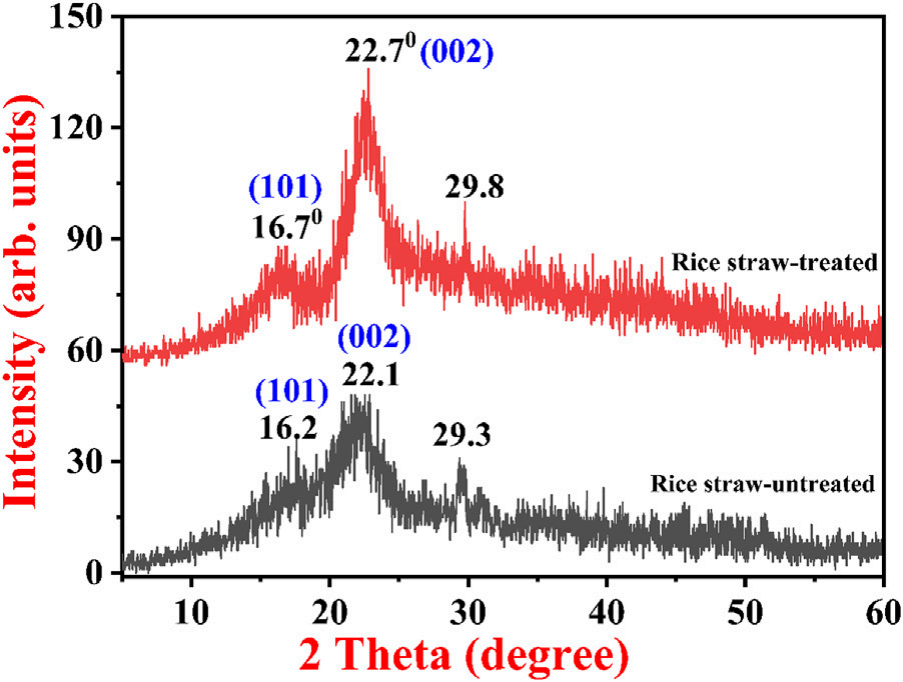
XRD spectra of untreated native and alkali-pretreated rice straw.

Figure 2 exhibits a typical XRD pattern of untreated and treated RS. The untreated RS shows small-intensity amorphous peaks starting from 10° to 15° and an intermediate intensity peak from 20° to 25°, which indicates amorphous carbon-containing compounds like lignin, hemicellulose, and cellulose. In addition to this, there is a small intensity peak in the untreated RS at 29.3° due to the quartz silicate peak, which is commonly present in RS. No other significant peaks were observed in the untreated RS sample. In the pretreated RS, the intensity of peaks at 16.7° (101) became more prominent, while another major peak was observed at 22.7° (002). This particular peak became more intense after the alkali treatment, which increased the crystallinity of the sample; that is, it became crystalline cellulose. In addition, there was a small peak at 29.8° due to the crystalline quartz/silicate peak. The major observation in both samples was that in the NaOH-treated RS, the intensity of the weak peaks significantly increased, resulting in enhanced peak resolution. The major peak starting from 19° to 25° increased drastically in the alkali-treated sample, which could be due to the exfoliation of the previously hidden active sites on the treated RS. From the XRD spectra, it is evident that the crystallinity changed in the substrate after pretreatment. One of the most significant factors that affect the hydrolysis of enzymes in lignocellulosic biomass is the crystallinity of the RS (AboDalam et al., 2022). After the pretreatment conditions were applied, the crystallite size increased, that is, it was 3.07 nm, whereas the crystallite size was only 2.71 nm in the native RS**.** The increased crystallite size may be due to the removal of external fibers, which leads to an increase in surface area, thus making cellulose more accessible to enzymes. Previously, Phuong et al. observed similar results for the untreated and NaOH-treated RS. Here the investigators obtained two peaks, one for amorphous cellulose and another for crystalline cellulose. In the NaOH-treated RS, degradation was observed in both the crystalline and amorphous forms of the cellulose. Furthermore, it was reported that major degradation was observed in the amorphous forms of cellulose and a small fraction in the crystalline cellulose. This could be because the crystalline form of cellulose is inert and harder to degrade or hydrolyze with NaOH than the amorphous form (Phuong et al., 2017).

#### 3.3.3 Surface morphology analysis by SEM

**FIGURE 3:**
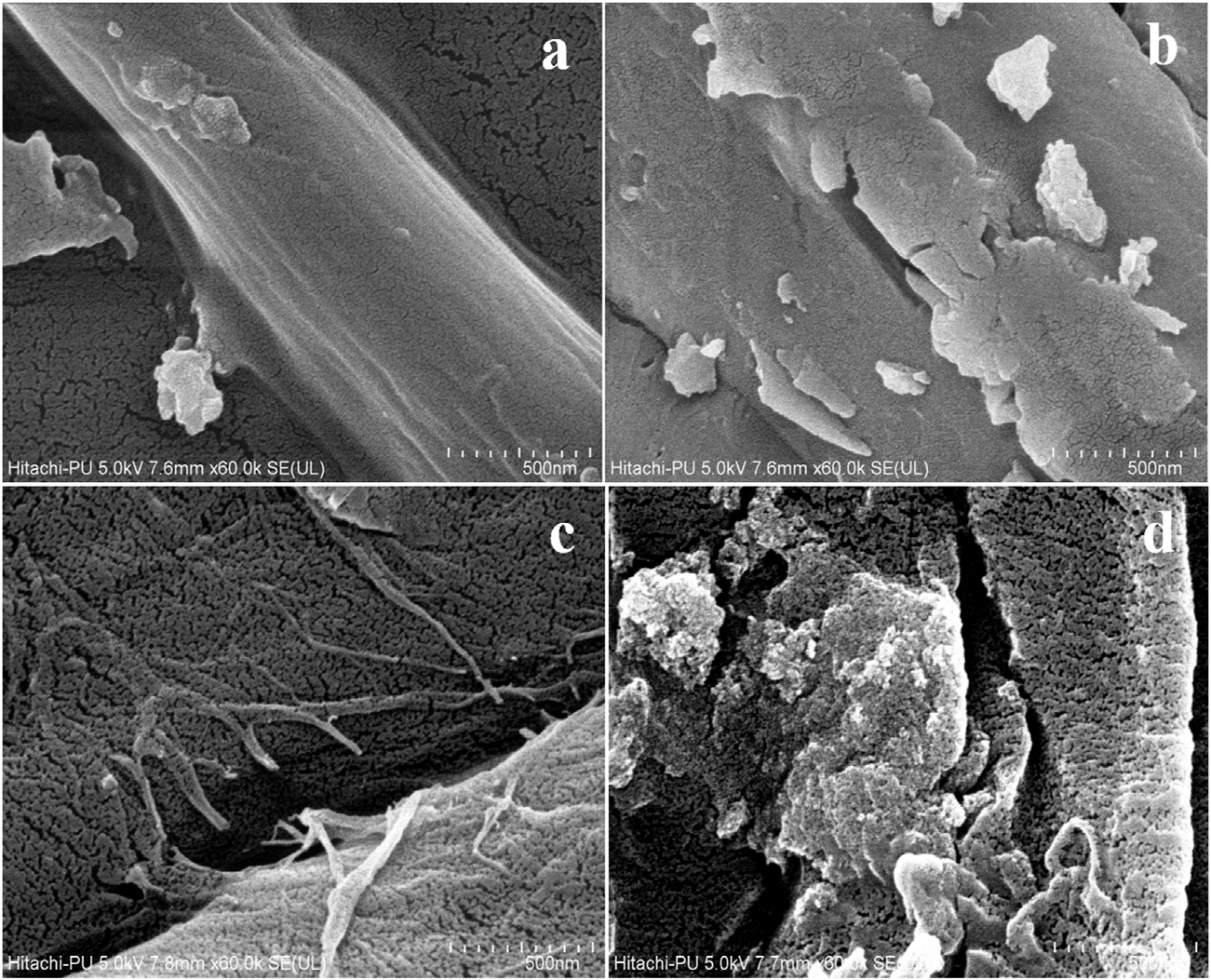
SEM micrographs of untreated rice straw **(A,B)** and alkali-treated rice straw **(C,D)**.

SEM was employed to investigate the surface morphology of fibers and the intricate surface structure of solid materials. The detailed SEM analysis showed the altered morphological changes in the alkali-treated sample. Figures 3A,B exhibit the well-organized fibrous structure and the intact surface in the native plant cell wall, thereby suggesting the presence of lignin coverage on the fibers. Figures 3C,D depict the significantly distorted, rough, and disorganized structure of the RS. The disintegration of the joined fibrous matrix and the formation of porosity may be due to lignin solubilization. The disruption in the plant cell wall occurs only after the pretreatment conditions with an increase in the external surface area, enhancing the enzymatic hydrolysis. Thus, it has been proved that alkali reduces the recalcitrant nature of the RS by dissolving the cellulose fibers, thereby enhancing cellulose accessibility to enzymes. Previously, Phitsuwan et al. (2017) and Feng et al. (2023) also obtained similar morphological results for RS. Phitsuwan treated the RS with an ammonia solution. The untreated RS shows an intact structure on the surface, indicating the covering of lignin on the surface. The RS treated with ammoniacal solution showed a distorted structure due to the solubilization of lignin from the surface of the RS.

### 3.4 SSF process

The ethanol concentration was calculated based on the glucose yield from RS. The highest ethanol concentration (21.45%) was obtained when alkali-pretreated rice RS was hydrolyzed with (2:1:1) enzyme loading, 20% solid loading, and fermentation for 1 week at 30°C and pH 4.5 with the yeast isolate *Saccharomyces cerevisiae* through the SSF process. This concentration was very much higher than that of the untreated biomass (3.67 g/L).

### 3.5 Ethanol analysis by (nuclear magnetic resonance spectroscopy)

**FIGURE 4:**
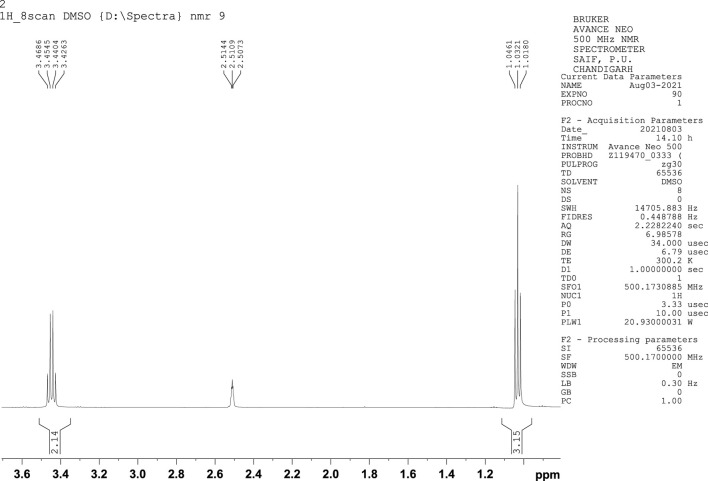
^1^H NMR spectra of ethanol.

**FIGURE 5:**
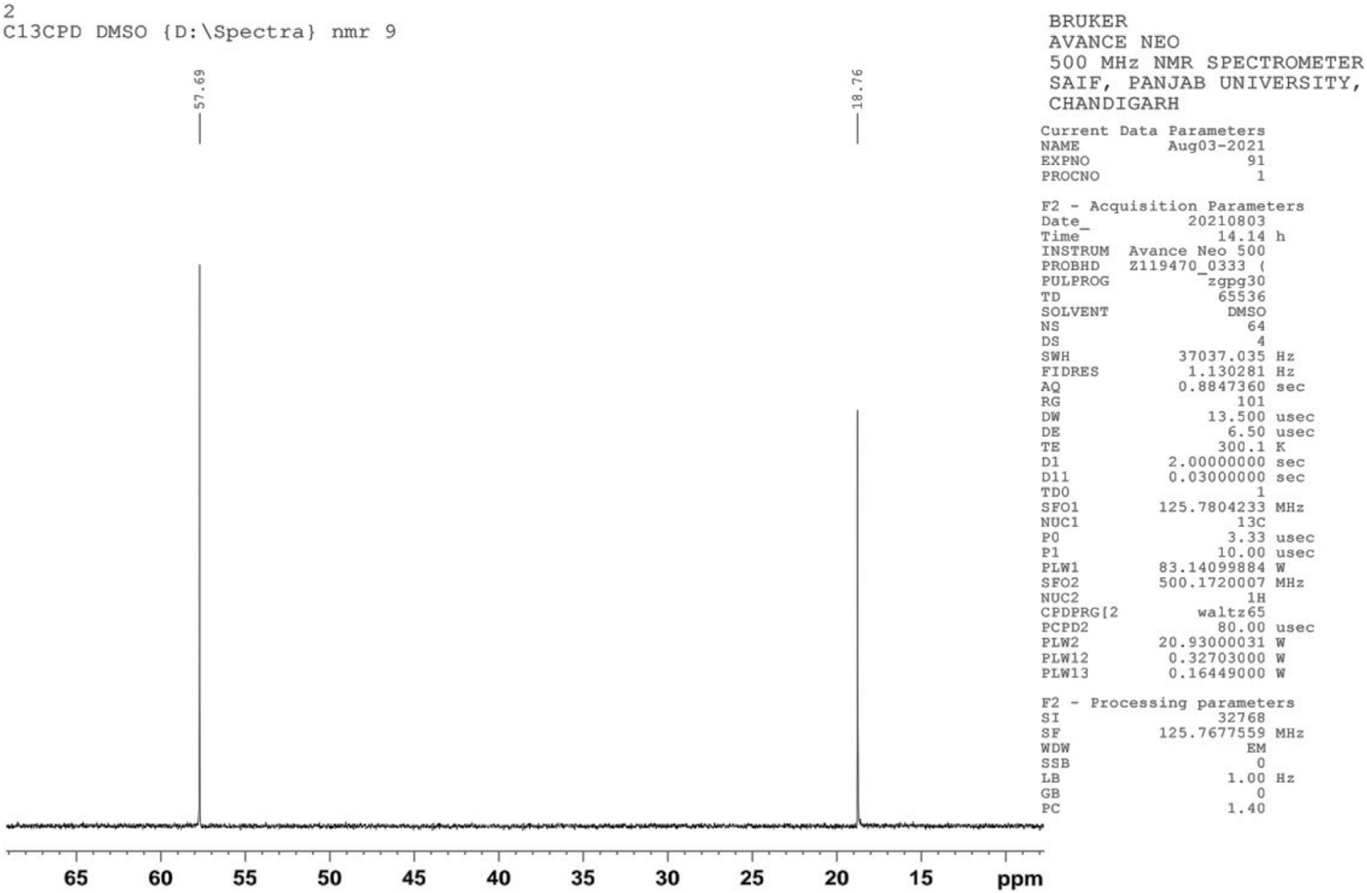
^13^C spectra of ethanol.

NMR is a significant method employed in the determination of the chemical structure of compounds. NMR has been used to investigate various aspects of bioethanol, like monomer units, cellular content, conformational analysis, monomer linkage sequence, co-polymer analysis, and bioethanol metabolic pathway studies. Determination of ethanol using ^1^H NMR spectroscopy, which offers many signals of different molecules in a single spectrum, continues to be a target in recent studies. The ^1^H NMR peaks of ethanol showed intensity ratios of 3:2:1. The proton nuclear magnetic resonance (^1^H NMR) spectrum exhibits a triplet corresponding to three protons at 1.03 ppm, which represents the terminal methyl proton, a triplet corresponding to one proton at 2.51 ppm corresponding to the hydroxyl group, and a quartet at 3.44 ppm due to the methylene protons (Figure 4). In the C-13 NMR spectrum of ethanol, only two chemical shift lines were observed, indicating the presence of only two different chemical environments of carbon atoms. The C-13 NMR spectrum showed one peak at 18.76 ppm, corresponding to a methyl carbon, and another at approximately 57.69 ppm, corresponding to a methylene carbon, (Figure 5). These are the characteristic features of ethanol.

## 4 Conclusion

The alkali-treated biomass caused a decrease in the cellulose crystallinity. The FTIR, XRD, and SEM analysis clearly showed the changes in functional groups, removal of lignin after applying NaOH treatment, and increase in the surface area, thereby enhancing the cellulose accessibility to hydrolysis, increasing the cellulose crystallinity, and distorting the fibrous structure. A fermentation efficiency of 21.45% ethanol was attained. These results indicate that alkali treatment using 4% NaOH could be effective for second-generation bioethanol production. Our investigation concludes that alkali pretreatment of RS provides a promising option for increasing enzymatic hydrolysis efficiency, making RS a suitable feedstock in bioethanol production. The bioethanol so obtained was eco-friendly and an alternative energy source.

## Data Availability

The original contributions presented in the study are included in the article/Supplementary Material; further inquiries can be directed to the corresponding authors.

## References

[B1] AboDalam H. Devra V. Ahmed F. K. Li B. Abd-Elsalam K. A. (2022). “Chapter 29 - rice wastes for green production and sustainable nanomaterials: An overview,” in Agri-waste and microbes for production of sustainable nanomaterials. Editors Abd-Elsalam K. A. Periakaruppan R. Rajeshkumar S. (Netherlands: Elsevier), 707–728. 10.1016/B978-0-12-823575-1.00009-3

[B2] Arun V. Perumal E. M. Prakash K. A. Rajesh M. Tamilarasan K. (2020). Sequential fractionation and characterization of lignin and cellulose fiber from waste rice bran. J. Environ. Chem. Eng. 8, 104124. 10.1016/j.jece.2020.104124

[B3] Bhattacharyya P. Bhaduri D. Adak T. Munda S. Satapathy B. S. Dash P. K. (2020). Characterization of rice straw from major cultivars for best alternative industrial uses to cutoff the menace of straw burning. Ind. Crops Prod. 143, 111919. 10.1016/j.indcrop.2019.111919

[B4] Bhattacharyya P. Bisen J. Bhaduri D. Priyadarsini S. Munda S. Chakraborti M. (2021). Turn the wheel from waste to wealth: Economic and environmental gain of sustainable rice straw management practices over field burning in reference to India. Sci. Total Environ. 775, 145896. 10.1016/j.scitotenv.2021.145896

[B5] Broda M. Yelle D. J. Serwańska K. (2022). Bioethanol production from lignocellulosic biomass—challenges and solutions. Molecules 27, 8717. 10.3390/molecules27248717 36557852 PMC9785513

[B6] De S. Mishra S. Poonguzhali E. Rajesh M. Tamilarasan K. (2020). Fractionation and characterization of lignin from waste rice straw: Biomass surface chemical composition analysis. Int. J. Biol. Macromol. 145, 795–803. 10.1016/j.ijbiomac.2019.10.068 31739019

[B7] Deng W. Feng Y. Fu J. Guo H. Guo Y. Han B. (2023). Catalytic conversion of lignocellulosic biomass into chemicals and fuels. Green Energy & Environ. 8, 10–114. 10.1016/j.gee.2022.07.003

[B8] Dimos K. Paschos T. Louloudi A. Kalogiannis K. G. Lappas A. A. Papayannakos N. (2019). Effect of various pretreatment methods on bioethanol production from cotton stalks. Fermentation 5, 5. 10.3390/fermentation5010005

[B9] Dsouza H. S. Sajankila S. P. Satyamoorthy K. (2012). Manipal laboratory manual for biotechnologists. Manipal: Manipal University Press.

[B10] Feng B. Liu J. Lu Z. Zhang M. Tan X. (2023). Study on properties and durability of alkali activated rice straw fibers cement composites. J. Build. Eng. 63, 105515. 10.1016/j.jobe.2022.105515

[B11] Fukagawa Naomi K. Ziska L. H. (2019). Rice: Importance for global nutrition. J. Nutr. Sci. Vitaminol. (Tokyo) 65, S2–S3. 10.3177/jnsv.65.S2 31619630

[B12] Goodman B. A. (2020). Utilization of waste straw and husks from rice production: A review. J. Bioresour. Bioprod. 5, 143–162. 10.1016/j.jobab.2020.07.001

[B13] Guleria P. Kaur S. Sidana A. Yadav S. K. (2022). Xylitol production from rice straw hemicellulosic hydrolysate by *Candida tropicalis* GS18 immobilized on bacterial cellulose-sodium alginate matrix. Biomass Convers. Biorefin 2022, 02986. 10.1007/s13399-022-02986-0

[B14] Hassan Md. K. Chowdhury R. Ghosh S. Manna D. Pappinen A. Kuittinen S. (2021). Energy and environmental impact assessment of Indian rice straw for the production of second-generation bioethanol. Sustain. Energy Technol. Assessments 47, 101546. 10.1016/j.seta.2021.101546

[B15] He Y. Pang Y. Liu Y. Li X. Wang K. (2008). Physicochemical characterization of rice straw pretreated with sodium hydroxide in the solid state for enhancing biogas production. Energy & Fuels 22, 2775–2781. 10.1021/ef8000967

[B16] Huang L. Z. Ma M. G. Ji X. X. Choi S. E. Si C. (2021). Recent developments and applications of hemicellulose from wheat straw: A review. Front. Bioeng. Biotechnol. 9, 690773. 10.3389/fbioe.2021.690773 34239863 PMC8258147

[B17] Kaur A. Kuhad R. C. (2019). Valorization of rice straw for ethanol production and lignin recovery using combined acid-alkali pre-treatment. Bioenergy Res. 12, 570–582. 10.1007/s12155-019-09988-3

[B18] Kaur J. Mankoo R. K. Chahal G. K. (2023). Synthesis of rice straw biopolymers based hydrogels and their use as media for growth of monocot (wheat) and dicot (moong bean) plants. Chem. Pap. 77, 2539–2555. 10.1007/s11696-022-02644-9

[B19] Khan M. F. S. Akbar M. Xu Z. Wang H. (2021). A review on the role of pretreatment technologies in the hydrolysis of lignocellulosic biomass of corn stover. Biomass Bioenergy 155, 106276. 10.1016/j.biombioe.2021.106276

[B20] Khantibongse P. Ratanatamskul C. (2023). Insight into pathway of monosaccharide production from integrated enzymatic hydrolysis of rice straw waste as feed stock for anaerobic digestion. Sci. Rep. 13, 148. 10.1038/s41598-023-27398-6 36600032 PMC9813138

[B21] Kumari D. Singh R. (2022). Rice straw structure changes following green pretreatment with petha wastewater for economically viable bioethanol production. Sci. Rep. 12, 10443. 10.1038/s41598-022-14627-7 35729221 PMC9213452

[B22] Laca A. Laca A. Díaz M. (2019). “Chapter 8 - hydrolysis: From cellulose and hemicellulose to simple sugars,” in Second and third generation of feedstocks. Editors Basile A. Dalena F. (Netherlands: Elsevier), 213–240. 10.1016/B978-0-12-815162-4.00008-2

[B23] Louis A. C. F. Venkatachalam S. Gupta S. (2022). Innovative strategy for rice straw valorization into nanocellulose and nanohemicellulose and its application. Ind. Crops Prod. 179, 114695. 10.1016/j.indcrop.2022.114695

[B24] Luo H. Gao L. Xie F. Shi Y. Zhou T. Guo Y. (2022). A new l-cysteine-assisted glycerol organosolv pretreatment for improved enzymatic hydrolysis of corn stover. Bioresour. Technol. 363, 127975. 10.1016/j.biortech.2022.127975 36122842

[B25] Luo H. Shi Y. Xie F. Zhou T. Gao L. Yang R. (2023). Efficient co-production of fermentable sugars and biobutanol from corn stover based on a novel butyric acid pretreatment strategy. Ind. Crops Prod. 191, 115976. 10.1016/j.indcrop.2022.115976

[B26] Malik K. Sharma P. Yang Y. Zhang P. Zhang L. Xing X. (2022). Lignocellulosic biomass for bioethanol: Insight into the advanced pretreatment and fermentation approaches. Ind. Crops Prod. 188, 115569. 10.1016/j.indcrop.2022.115569

[B27] Mankar A. R. Pandey A. Modak A. Pant K. K. (2021). Pretreatment of lignocellulosic biomass: A review on recent advances. Bioresour. Technol. 334, 125235. 10.1016/j.biortech.2021.125235 33957458

[B28] Mankoo R. K. Kaur J. Chahal G. K. (2023). Characterization of rice straw lignin phenolics and evaluation of their role in pollen tube growth in Cucurbita pepo L. Nat. Prod. Res. 2023, 1–6. 10.1080/14786419.2023.2225126 37322891

[B29] Md Salim R. Asik J. Sarjadi M. S. (2021). Chemical functional groups of extractives, cellulose and lignin extracted from native Leucaena leucocephala bark. Wood Sci. Technol. 55, 295–313. 10.1007/s00226-020-01258-2

[B30] Melendez J. R. Mátyás B. Hena S. Lowy D. A. El Salous A. (2022). Perspectives in the production of bioethanol: A review of sustainable methods, technologies, and bioprocesses. Renew. Sustain. Energy Rev. 160, 112260. 10.1016/j.rser.2022.112260

[B31] Ningthoujam R. Dhingra H. K. (2021). Bioethanol over production from second generation feedstock using rice straw as lignocellulosic waste. Curr. Trends Biotechnol. Pharm. 15, 14–18. 10.5530/ctbp.2021.6.4

[B32] Ningthoujam R. Dhingra H. K. (2022). Production of hydrolytic cellulase enzyme by isolate Aspergillus flavus OR and Trichoderma reseei using rice straw as the feedstock material. Available at: http://www.ncbi .

[B33] Phitsuwan P. Permsriburasuk C. Baramee S. Teeravivattanakit T. Ratanakhanokchai K. (2017). Structural analysis of alkaline pretreated rice straw for ethanol production. Int. J. Polym. Sci. 2017, 1–9. 10.1155/2017/4876969

[B34] Phuong N. T. M. Hoang P. H. Dien L. Q. Hoa D. T. (2017). Optimization of sodium sulfide treatment of rice straw to increase the enzymatic hydrolysis in bioethanol production. Clean. Technol. Environ. Policy 19, 1313–1322. 10.1007/s10098-016-1329-2

[B35] Prajapati B. P. Kango N. (2023). Rice straw saccharification using cellulolytic cocktail from Aspergillus tubingensis and structure alterations studies of the wall polymer. Biomass Convers. Biorefin 13, 961–975. 10.1007/s13399-020-01237-4

[B36] Ren J. Yu P. Xu X. (2019). Straw utilization in China-status and recommendations. Sustain. Switz. 11, 1762. 10.3390/su11061762

[B37] Sahoo D. K. Stork J. DeBolt S. Maiti I. B. (2013). Manipulating cellulose biosynthesis by expression of mutant Arabidopsis*proM24:CESA3ixr1-2*gene in transgenic tobacco. Plant Biotechnol. J. 11, 362–372. 10.1111/pbi.12024 23527628

[B38] Sanni A. Olawale A. S. Sani Y. M. Kheawhom S. (2022). Sustainability analysis of bioethanol production from grain and tuber starchy feedstocks. Sci. Rep. 12, 20971. 10.1038/s41598-022-24854-7 36470926 PMC9722859

[B54] Sindhu R. Binod P. Janu K. U. Sukumaran R. K. Pandey A. (2012). Organosolvent pretreatment and enzymatic hydrolysis of rice straw for the production of bioethanol. World J. Microbiol. Biotechnol. 28, 473–483. 10.1007/s11274-011-0838-8 22806842

[B39] Singh G. Gupta M. K. Chaurasiya S. Sharma V. S. Pimenov D. Y. (2021). Rice straw burning: A review on its global prevalence and the sustainable alternatives for its effective mitigation. Environ. Sci. Pollut. Res. 28, 32125–32155. 10.1007/s11356-021-14163-3 33934301

[B40] Sonwani R. Gupta S. B. Soni R. (2020). Production of bioethanol from biodegraded alkali pretreated rice straw. Vegetos 33, 128–134. 10.1007/s42535-019-00089-2

[B41] Sreejith R. P. Sankar M. Sukumaran R. K. Savithri S. (2022). Rapid estimation of the chemical composition of rice straw using FTIR spectroscopy: A chemometric investigation. Biomass Convers. Biorefin 2022, 03508. 10.1007/s13399-022-03508-8

[B42] Sunkar B. Bhukya B. (2022). An approach to correlate chemical pretreatment to digestibility through biomass characterization by SEM, FTIR and XRD. Front. Energy Res. 10, 802522. 10.3389/fenrg.2022.802522

[B43] Taghizadeh-Alisaraei A. Motevali A. Ghobadian B. (2019). Ethanol production from date wastes: Adapted technologies, challenges, and global potential. Renew. Energy 143, 1094–1110. 10.1016/j.renene.2019.05.048

[B44] Takano M. Hoshino K. (2018). Bioethanol production from rice straw by simultaneous saccharification and fermentation with statistical optimized cellulase cocktail and fermenting fungus. Bioresour. Bioprocess 5, 16. 10.1186/s40643-018-0203-y

[B45] Tan J. Li Y. Tan X. Wu H. Li H. Yang S. (2021). Advances in pretreatment of straw biomass for sugar production. Front. Chem. 9, 696030. 10.3389/fchem.2021.696030 34164381 PMC8215366

[B46] Van Hung N. Maguyon-Detras M. C. Migo M. V. Quilloy R. Balingbing C. Chivenge P. (2020). “Rice straw overview: Availability, properties, and management practices,” in Sustainable rice straw management. Editors Gummert M. Van Hung N. Chivenge P. Douthwaite B. (Berlin, Germany: Springer International Publishing), 1–13. 10.1007/978-3-030-32373-8_1

[B47] Xin F. Geng A. (2010). Horticultural waste as the substrate for cellulase and hemicellulase production by Trichoderma reesei under solid-state fermentation. Appl. Biochem. Biotechnol. 162, 295–306. 10.1007/s12010-009-8745-2 19707729

[B48] Yadav V. K. Amari A. Wanale S. G. Osman H. Fulekar M. H. (2023). Synthesis of floral-shaped nanosilica from coal fly ash and its application for the remediation of heavy metals from fly ash aqueous solutions. Sustainability 15, 2612. 10.3390/su15032612

[B49] Yadav V. K. Fulekar M. H. (2019). Green synthesis and characterization of amorphous silica nanoparticles from fly ash. Mater. Today Proc. 18, 4351–4359. 10.1016/j.matpr.2019.07.395

[B50] Yadav V. K. Gupta N. Kumar P. Dashti M. G. Tirth V. Khan S. H. (2022). Recent advances in synthesis and degradation of lignin and lignin nanoparticles and their emerging applications in nanotechnology. Materials 15, 953. 10.3390/ma15030953 35160893 PMC8838035

[B51] Yan H. Zhang Q. Wang Y. Cui X. Liu Y. Yu Z. (2023). Rice straw as microalgal biofilm bio-carrier: Effects of indigenous microorganisms on rice straw and microalgal biomass production. J. Environ. Manage 341, 118075. 10.1016/j.jenvman.2023.118075 37141712

[B52] Yang H. Yan R. Chen H. Zheng C. Lee D. H. Liang D. T. (2006). In-depth investigation of biomass pyrolysis based on three major components: Hemicellulose, cellulose and lignin. Energy & Fuels 20, 388–393. 10.1021/ef0580117

[B53] Zhao Y. Shakeel U. Saif Ur Rehman M. Li H. Xu X. Xu J. (2020). Lignin-carbohydrate complexes (LCCs) and its role in biorefinery. J. Clean. Prod. 253, 120076. 10.1016/j.jclepro.2020.120076

